# Supplementary material for: Exploring the Longitudinal Stability of Food Neophilia and Dietary Quality and Their Prospective Relationship in Older Adults: A Cross-Lagged Panel Analysis
Source: Nutrients. 2023 Mar 1;15(5):1248. doi: 10.3390/nu15051248 (PMC10005135; doi:10.3390/nu15051248)
Supplement: Supplementary file 1 [file nutrients-15-01248-s001.zip › nutrients-2228115-supplementary.pdf]

Table S1. Fit indices for invariance tests of the VARSEEK across T1 and T2 (N = 960).

Model	CFI	RMSEA	SRMR	Model comparison	ΔCFI	ΔRMSEA	ΔSRMR
M1: Configural	.968	.061	.032				
M2: Metric	.967	.060	.035	M1 vs. M2	–.001	–.001	.003
M3: Scalar	.965	.060	.037	M2 vs. M3	–.002	<.001	.002
M4: Residual	.963	.059	.039	M3 vs. M4	–.002	–.001	.002

CFI = comparative fit index; RMSEA = root mean square error of approximation; SRMS = standardized root mean square residual.
